# Effects of Combined Repeated Sprint and Large-Sided Game Training on Physical Performance in Elite U20 Soccer Players: A Randomised Controlled Trial

**DOI:** 10.3390/sports13110394

**Published:** 2025-11-05

**Authors:** Mehdi Ben Brahim, Bekir Erhan Orhan, Hussain Yasin, Shaher A. I. Shalfawi

**Affiliations:** 1 Health and Physical Education Department, Prince Sultan University, Riyadh 11586, Saudi Arabia; hyasin@psu.edu.sa; 2 Faculty of Sports Sciences, Istanbul Aydın University, 34295 Istanbul, Turkey; bekirerhanorhan@aydin.edu.tr; 3 Department of Education and Sports Science, University of Stavanger, 4036 Stavanger, Norway; shaher.shalfawi@uis.no

**Keywords:** repeated sprint training, large-sided soccer games, agility, vertical jump

## Abstract

Background: The purpose of the present study was to investigate the impact of a combined Repeated Sprint Training (RST) with Large-Sided Soccer Games (LSSG) on soccer players’ physical performance indicators. Methods: A randomised controlled trial protocol was designed and implemented to examine the effects of an 8-week training programme on the physical performance of U20 national team soccer players. Participants were randomly assigned after matching them based on their pre-test results from a 30 m sprint to one of two groups: an experimental group (EG; *n* = 16) and a control group (CG; *n* = 10). The EG took part in two extra training sessions per week, which included RST and LSSG, whereas the CG stuck to their usual training routine. Sprint, Repeated sprint ability (RSA), vertical jump, the New Multi-Change of Direction Agility Test (NMAT), and the 15 m ball dribbling agility test performances were assessed. Results: The main findings from this study indicate that the EG showed statistically significant improvements in short sprint performance (5 m), vertical jump height (SJ and CMJ), agility (NMAT), RSA, and fatigue tolerance, with moderate to large effect sizes. The CG showed no statistically significant changes, though some small to moderate effect sizes were observed. Conclusions: The findings suggest that this hybrid method has the potential to produce improvements in specific performance domains, particularly agility and fatigue tolerance, beyond what may be expected from regular soccer training alone.

## 1. Introduction

The soccer game is characterised by rapid transitions, explosive efforts, and complex tactical exchanges that demand athletes to perform at higher levels of physical and cognitive ability [1,2,3,4]. Especially at the elite youth level, the development of key physical traits (including sprint speed, jumping ability, and agility among under-20 (U20) players) influences performance growth and successful progression to senior-level competition [5,6,7]. Among other actions, these attributes directly impact rapid changes in direction, defensive recoveries, one-to-one duels, and sprints preceding scoring. Recent evidence underscores the importance of agility and change-of-direction skills as critical determinants of performance in youth soccer, making them essential targets for training interventions [5,7].

Creating scoring chances or preventing opponents from advancing depends on sprinting short distances [8,9]. Contesting headers requires jumping ability, especially vertical jumps; agility helps players change direction rapidly in response to unexpected game situations [5,7]. There is a growing need to use training programmes that focus on these physical qualities and replicate the intensity and specificity of match scenarios, as the sport’s physical demands evolve [10,11,12]. A well-established method to enhance anaerobic performance and neuromuscular function is repeated sprint training (RST) [12,13]. The RST aims to improve an athlete’s ability to perform multiple high-intensity efforts with minimal performance decline by executing a series of short sprints with limited recovery [12,13]. It has been shown that RST improve motor unit recruitment, lactate buffering, and increases phosphocreatine resynthesis [14,15].

Large-sided soccer games (LSSG), which involve larger playing areas and more players than small-sided games, have been shown to produce a greater overall physical load while maintaining tactical and technical engagement [16,17]. The LSSG is an effective method for mimicking match play during training [18], placing simultaneous stress on both aerobic and anaerobic systems. This helps enhance endurance, acceleration, deceleration, and positioning awareness [12,13]. Furthermore, technical execution, spatial awareness, and real-time decision-making add a cognitive and strategic dimension often missing in isolated drills [18,19]. Although RST and LSSG are both effective, limited research has examined the potential benefits of combining these modalities into a single training programme [6,20]. The core idea is that each approach offers distinct yet complementary stimuli: RST provides controlled, intense loading targeting specific physical systems [10,12], while LSSG simulate match-like efforts in dynamic environments [20,21]. A combined protocol presents an integrated method that increases the likelihood of transferring gains from RST to actual match conditions.

Soccer players under 20 years old are approaching physical maturity while also improving their tactical awareness and sport-specific skills [22,23]. Therefore, training interventions should be designed to maximise gains without hindering skill development [24,25]. Combining RST with LSSG could serve as a practical, time-efficient method for addressing these needs. Additionally, integrating game-based elements into conditioning programmes can help maintain player motivation, reduce boredom, and create a more engaging and sustainable training environment [18,19]. By incorporating physical development within gameplay, coaches can directly promote meaningful improvements in player performance [10,12].

Recent evidence shows that Small-Sided Games (SSG) and RST drive partly distinct adaptations relevant to soccer. The SSG integrates technical/decision constraints with aerobic–anaerobic stresses, whereas RST targets brief, high-intensity neuromuscular efforts [26,27]. Building on these findings, combining game-based work with a targeted sprint is expected to provide complementary stimuli and facilitate better transfer to gameplay. Furthermore, the LSSG offers a similar stimulus to the actual gameplay compared to SSG (i.e., greater distance covered per minute during LSSG than during SSG, and a higher rate of accelerations and decelerations during SSG than during LSSG).

Therefore, the purpose of this randomised controlled study was to address these evolving practices by implementing a combined RST with LSSG training programme on U20 national team soccer players. We hypothesised that adding a combined RST and LSSG twice a week would yield greater improvements in agility, sprint performance, and vertical jump performance than regular soccer training alone.

## 2. Materials and Methods

### 2.1. Study Design

This randomised controlled trial (RCT) examined the effects of an 8-week programme of combined RST and LSSG on various physical performance tests in U20 national team soccer players. Participants were randomly allocated to one of two groups: an experimental group (EG) and a control group (CG). The EG undertook two additional training sessions per week involving RST and LSSG, while the CG continued with their usual training routine.

### 2.2. Participants

Initially, all U-20 national team players (*n* = 38) were recruited and agreed to participate in this study, including four goalkeepers; however, only the outfield players were included in the analyses. Participants were randomly assigned after matching them based on their pre-test performance on the 30 m sprint to one of two groups (EG *n* = 16; and CG *n* = 16). During the intervention, six from the CG dropped out of the study. To ensure that the assumption of homogeneity of variance was not violated by participant dropout, a comprehensive preliminary analysis of pre-test scores was conducted across groups. The results, which are detailed in the “Supplementary Materials: Tests of Homogeneity of Variances,” showed no significant violations of the homogeneity of variance assumption. Hence, twenty-six elite U20 male soccer players, with an average age of 19.1 ± 0.7 years, a height of 181 ± 0.6 cm, a body mass of 75.9 ± 4.9 kg, and a body fat percentage of 12.0 ± 0.8%, completed this study (i.e., EG (*n* = 16), aged 19.3 ± 0.7 years, height 182 ± 0.7 cm, body mass 75.0 ± 5.2 kg, and body fat 12.7 ± 0.8%, and CG (*n* = 10), aged 18.9 ± 0.6 years, height 180 ± 0.6 cm, body mass 77.9 ± 3.9 kg, and body fat 11.8 ± 0.6%). Before the start of the experimental phase, all participants were informed about the study’s aims and potential risks and provided written informed consent. The study was conducted in accordance with the ethical principles outlined in the Declaration of Helsinki. Players who had sustained injuries within the previous three months or had joined the team within the past six months were excluded from participation.

### 2.3. Experimental Design

**Figure 1:**
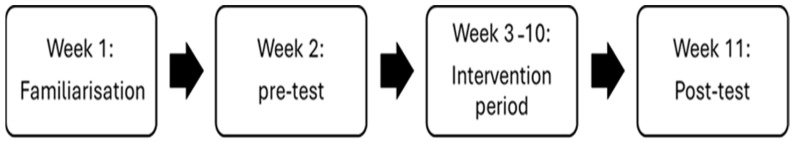
The experimental design.

The intervention was conducted during the in-season mesocycle. This study lasted eleven weeks, with one week dedicated to training familiarisation, one week for baseline testing, eight weeks of intervention, and one week of post-testing (Figure 1).

#### 2.3.1. Training Characteristics

The training mesocycle for both the CG and the EG lasted 8 weeks, comprising 40 training days (Monday and Sunday were not considered training days). Over this period, athletes participated in five official matches. Each week included approximately 7 to 8 training sessions (i.e., two on Tuesday, Wednesday, and Thursday; and one on Friday and Saturday), resulting in an average of around 60 throughout the mesocycle. The total training volume ranged from 10 to 12 h per week. Additionally, four days were allocated for recovery across the eight weeks.

#### 2.3.2. Weekly Training Design

Both groups followed the same regular training programme, with two additional sessions for the EG on Tuesdays and Thursdays (Table 1).

#### 2.3.3. Combined RST and LSSG Training Programme

The RST programme involved progressive overload from week 1 to 6, followed by two weeks of unloading (Table 2). The LSSG was played in a 7-vs.-7 format on a 68 m by 54 m pitch. The RST and the LSSG were performed in the same training session, with RST conducted first, followed by LSSG. The sessions took place on natural grass fields during the afternoon. All players included in the analyses completed more than 95% of the scheduled sessions, and no injuries or adverse events were reported during the intervention. The Game included two bouts without using the offside rule across the larger pitch, and no throw-ins were permitted. Players were not allowed to stop or walk during the Game, encouraging continuous movement. Three variations in LSSG were used: a 1-ball touch limit, a 2-ball touch limit, and a free play version. The Game aimed to maintain ball possession, supported by four external support players. The training comprised two sets of three 4 min bouts, each followed by 3 min of active recovery.

### 2.4. Study Measurements

#### 2.4.1. Anthropometric Measurements

Body mass was measured using a calibrated digital scale (seca 813, Seca Instruments Ltd., Hamburg, Germany) and height were measured using a stadiometer (seca 217, Seca Instruments Ltd., Hamburg, Germany). Skinfold thickness was measured at four anatomical sites using Harpenden callipers (British Indicators Ltd., St. Albans, UK), and body fat percentage (BF%) was then calculated according to Durnin and Womersley [28].

#### 2.4.2. Vertical Jump Performance

Vertical jump performance was evaluated using the Opto Jump system (Microgate SARL, Bolzano, Italy). Participants performed both the squat jump (SJ) and the countermovement jump (CMJ) according to standardised protocols [29]. After one familiarisation trial, each type of jump was repeated twice, with a two-minute rest between attempts. The highest score from the two trials was used for analysis.

#### 2.4.3. Sprint Performance Tests

A standard warm-up included 5 min of light jogging, dynamic stretching, and two submaximal sprint and jump trials to ensure participants were prepared adequately before testing. Sprint performance was evaluated over distances of 5, 10, and 30 m, with participants starting from a standing position 0.3 m behind the starting line. Participants initiated sprints themselves when they felt ready, and they were instructed to perform each effort at maximum intensity. Sprint times were recorded using waist-high photocell timing gates (HL3-1x Wireless Photocell, TAG Heuer Professional Timing, La Chaux-de-Fonds, Switzerland), with an accuracy of 0.01 s. After one familiarisation trial, each participant completed two timed sprints, separated by a five-minute rest period. The fastest time of the two attempts was used for analysis.

#### 2.4.4. New Multi-Change of Direction Agility Test (NMAT)

**Figure 2:**
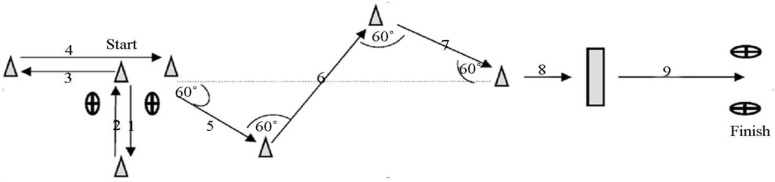
Schematic representation of the NMAT. (1) 2.5 m; (2) 2.5 m; (3) 2.5 m; (4) 3 m; (5) 2 m; (6) 4 m; (7) 2 m; (8) 1.5 m; (9) 5 m.

In the NMAT test [30], players’ velocities during a 25 m agility run were measured using waist-high photocell timing gates (HL3-1x Wireless Photocell, TAG Heuer Professional Timing). The test began with the athlete performing a 2.5 m lateral displacement, followed by a return over the same distance back to the starting point. From there, the player ran backwards for 2.5 m, then immediately transitioned into a 3 m forward sprint. Next, the athlete performed a 1 m change in direction, followed by a 1.5 m straight run, and then crossed a 0.5 m-high barrier (fence). The test concluded with a final 5 m linear sprint (Figure 2).

#### 2.4.5. Agility Test: 15 m Ball Dribbling, as Described by Mujika et al. [31]

**Figure 3:**
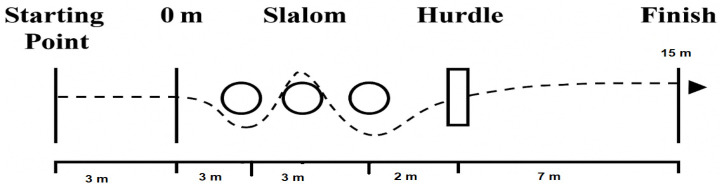
15 m ball dribbling, as described by Mujika et al. [31].

Five minutes after completing the NMAT, players performed the Ball-15 m test, which involved dribbling a ball for 15 m. During the slalom section, athletes maintained control of the ball as they navigated the agility course. At the hurdle, the ball was kicked underneath as the player jumped over it. After clearing the hurdle, the player kicked the ball towards one of two small goals positioned diagonally, 7 m to the left and right of the hurdle. The test concluded with a sprint to the finish line. Each player performed two maximum-effort attempts, separated by three minutes of passive recovery. The highest velocity achieved was recorded for analysis (Figure 3).

#### 2.4.6. Repeated Sprint Test

Participants started each sprint from a standing position, 50 cm behind the start/finish line, marked by a photocell (time zero). They sprinted 20 m in a straight line, touched a designated line with one foot, turned around, and ran back to the starting point, crossing the same photocell to record their finish time. After each sprint, participants had a 20 s passive rest period. Verbal cues were given at the 10 s and 15 s marks to help them prepare for the next sprint. At around 17 s, the test leader called out “ready”, followed by “go” at 20 s to signal the start of the next sprint. This cycle was repeated until all six sprints were completed, according to the protocol by Impellizzeri et al. [32]. Next, the Repeated Sprint Ability (RSA) test was performed, comprising six 40 m sprints (20 m out and back), with 20 s of passive rest between each sprint. Sprint times were recorded, and the fatigue index was calculated using the method described by Fitzsimons et al. [29] (i.e., Fatigue index = (∑ times/(t best × n° of sprints) × 100) – 100 = %).

### 2.5. Statistical Analyses

The collected data were transferred to a PC running Microsoft Windows 11 for further analysis. The normality of the data was assessed using the Shapiro–Wilk test in IBM SPSS Statistics for Windows, Version 25 (Armonk, NY, USA: IBM Corp.). To evaluate differences within groups, a paired *t*-test was conducted on each measured variable using GraphPad Prism version 6.00 for Windows (GraphPad Software, La Jolla, CA, USA). For the within-group differences, results were presented as mean difference, standard error of the difference, 95% Confidence Interval (95% CI) with the effect size (Cohen d) calculated and defined as small when *d* = 0.2–0.49, medium when *d* = 0.5–0.79 and large when *d* ≥ 0.8 [33]. Furthermore, between-groups differences were assessed using an independent-samples *t*-test. They were reported as mean difference in the difference from pre-to-post-test, standard error of the difference, 95% Confidence Interval (95% CI) with the effect size (Hedges’ g) calculated and defined as small when *g* = 0.2–0.49, medium when *g* = 0.5–0.79 and large when *g* ≥ 0.8 [33]. The alpha level for statistical significance was set to *p* ≤ 0.05 for all statistical examinations.

## 3. Results

Within-group analysis of the EG (Table 3) revealed statistically significant improvements in start and acceleration over 5 m (*p* = 0.003, *d* = 0.87), SJ height (*p* = 0.029, *d* = 0.62), CMJ height (*p* < 0.001, *d* = 0.85), NMAT performance (*p* = 0.013, *d* = 1.02), and RSA times (*p* < 0.001, *d* = 1.99). Additionally, fatigue tolerance improved (*p* < 0.001, *d* = 2.47).

The CG (Table 4) did not exhibit any statistically significant improvements across the measured variables (all *p* > 0.05). However, small to moderate effect sizes were observed in CMJ (*d* = 0.78), RSA FI (*d* = 0.85), and RSA times (*d* = 0.59).

Between-group comparisons (Table 5) revealed that the EG experienced statistically significantly greater improvements than the CG in the NMAT (*p* = 0.002, *g* = 1.34), RSA (*p* = 0.015, *g* = 1.02), and FI (*p* = 0.013, *g* = 1.05). These effects were large, as indicated by the *g*-values.

## 4. Discussion

This study aimed to investigate the effect of combining RST with LSSG on the physical performance of elite U20 male soccer players. Collectively, changes favoured the EG in agility (NMAT), repeated sprint ability (mean RSA time) and fatigue index, consistent with task-relevant neuromuscular adaptations elicited by the combined programme (see Table 3 for within-group changes and Table 5 for between-group effects). These patterns align with the task demands emphasised in the intervention and suggest that integrating brief, high-intensity RST blocks into LSSG-based microcycles can support agility and fatigue resistance in elite youth players.

The New Multi-Change of Direction Agility Test (NMAT) revealed that one of the most significant outcomes of the intervention was a substantial improvement in agility performance. This improvement demonstrates that the training programme effectively improved players’ ability to perform complex movements involving quick acceleration, deceleration, and changes in direction [4,5,7]. Such movements are fundamental in soccer, where players must constantly adapt to evolving game situations. The dynamic and reactive nature of LSSG likely has contributed to agility gains by exposing players to match-like movement constraints [16,20,21]. When combined with the explosive loading provided by RST, the resulting adaptations are consistent with improved neuromuscular coordination [14,15,34]. Because no cognitive or tactical measures were collected, these mechanisms should be considered hypothetical.

Dribbling agility (15 m) showed no apparent improvement. Beyond the need for ball-specific technical content, a ceiling effect is plausible in elite U20 players who already complete substantial ball-drill volumes during regular team practices and competition; under such conditions, additional physical gains may not readily transfer to this skill test within an 8-week mesocycle [35,36,37,38,39].

Another significant finding was a substantial improvement in repeated sprint ability (RSA), particularly in average sprint time and fatigue resistance. These improvements appear to have enhanced the players’ anaerobic capacity and their ability to recover quickly between high-intensity efforts [10,11,12]. This is crucial in soccer, where players must engage in frequent bouts of sprinting with brief recovery periods. Integrating RST into the LSSG allows players to simulate game-like demands in a controlled setting, leading to adaptations that transfer to real matches [17,20,21].

Vertical jump performance, evaluated by both SJ and CMJ, also showed marked improvements. This improvement likely reflects gains in lower-body power and neuromuscular activation [40,41], findings consistent with recent evidence on neuromuscular coordination [14,15]. However, the more moderate nature of these gains may be due to the training intervention’s focus on horizontal power and sprinting mechanics rather than vertical force production. Nonetheless, improvements in jump performance are valuable, particularly in aerial duels and explosive movements such as tackling, goal attempts, and defensive clearances [42,43,44,45].

Interestingly, improvements in short sprint acceleration over 5 m were statistically significant but longer sprints over 10 and 30 m did not show the same level of change. This could be due to the intervention RSA distance of 20 m, which was effective in improving start and acceleration speed—a skill crucial in soccer [9,39,40] and a key determinant of overall sprint performance. However, the lack of notable improvements in longer-distance sprints could be attributed to the nature of the training drills (Table 2), which emphasised rapid initiation and deceleration rather than maximal velocity phases [8,9,46].

LSSG may confer context-specific benefits—such as tactical spacing, team coordination, and in-game decision-making—by situating physical work within realistic match constraints [12,13,47]. These potential advantages could help contextualise physical adaptations during training. Nonetheless, because no cognitive or tactical outcomes were collected, these interpretations are speculative and should be tested in future work.

## 5. Conclusions

In elite U20 soccer, adding a combined RST and LSSG to the weekly microcycle appears feasible and aligns with agility and repeated sprint demands. This reinforces the principle of training specificity, underscoring the need for targeted anaerobic conditioning to achieve performance gains beyond those from conventional soccer training alone. This implies that while traditional training contributes to the maintenance of baseline capacities, it may not be sufficient to enhance explosive performance elements, such as agility or fatigue tolerance, which were notably better in the EG. This integrated training approach, therefore, represents a promising model that blurs the boundaries between conditioning and tactical development. Future work should use load-matched designs with objective monitoring (e.g., GPS, RPE, heart rate) and, where relevant, ball-skill outcomes to isolate modality-specific effects and clarify generalizability across standards and age groups.

### Limitations

Groups differed in total training volume due to two additional weekly sessions in the experimental group; thus, the effects may partially reflect a greater overall load rather than the type of training per se. The sample size was modest, assessors were not blinded, and no external or internal load monitoring (e.g., GPS, RPE, HR) was implemented during RST or LSSG, limiting the ability to infer dose–response relationships. Future studies are advised to include at least session-RPE or ball-in-play time in future combined-method trials.

## Figures and Tables

**Table 1:** The training programme followed by the CG and the EG.

Day	Time	Training Programme (CG & EG)	Extra Sessions (EG)
Monday	AM		
	PM	Regeneration session	
Tuesday	AM	Conditioning training	Combined RST and LSSG
	PM	Technical and Tactical training + Game	
Wednesday	AM	Technical training (Day off every 2 weeks)	
	PM	Technical and tactical training + Game (Day off Every 2 weeks)	
Thursday	AM	Conditioning training	Combined RST and LSSG
	PM	Tactical and Technical training + Game	
Friday	AM		
	PM	Conditioning and Technical training + Game	
Saturday	AM		
	PM	Technical and Tactical training	
Sunday	AM		
	PM	Match	

**Table 2:** Repeated sprint training programme followed by the EG.

Week	Sets	Rest Between Repetitions	Rest Between Sets
Week 1	2 Sets of 6 × 20 m = 240 m 12 sprints	30 s	3 min
Week 2	3 Sets of 6 × 20 m = 360 m 18 sprints	30 s	3 min
Week 3	3 Sets of 8 × 20 m = 480 m 24 sprints	30 s	3 min
Week 4	3 Sets of 10 × 20 m = 600 m 30 sprints	30 s	3 min
Week 5	3 Sets of 12 × 20 m = 720 m 36 sprints	30 s	3 min
Week 6	4 Sets of 10 × 20 m = 800 m 40 sprints	30 s	3 min
Week 7	4 Sets of 8 × 20 m = 640 m 32 sprints	30 s	3 min
Week 8	3 Sets of 8 × 20 m = 480 m 24 sprints	30 s	3 min

**Table 3:** Pre vs. Post-test results of the EG (*n* = 16).

	Pre-Test (SD)	Post Test (SD)	Diff (SE)	95% CI of Diff.	*p*-Value	*d*-Value	95% CI (*d*)
5 m (s)	1.10 (0.07)	1.05 (0.05)	0.06 (0.01)	0.02 to 0.10	0.003	−0.87	−1.4–−0.4
10 m (s)	1.91 (0.10)	1.85 (0.09)	0.06 (0.03)	−0.04 to 0.16	0.482	−0.66	−1.4–0.9
30 m (s)	4.35 (0.29)	4.26 (0.18)	0.09 (0.10)	−0.24 to 0.43	>0.99	−0.39	−1.3–0.6
SJ (cm)	26.8 (4.3)	29.3 (3.8)	−2.5 (0.7)	−4.87 to −0.19	0.029	0.62	0.2–1.0
CMJ (cm)	30.1 (4.2)	33.6 (3.5)	−3.4 (0.6)	−5.33 to −1.55	<0.001	0.85	0.4–1.3
15 m (ball) (M/s)	4.86 (0.40)	4.71 (0.38)	0.15 (0.14)	−0.30 to 0.60	0.968	−0.39	−1.2–0.4
NMAT (s)	9.79 (0.45)	9.39 (0.30)	0.37 (0.09)	0.07 to 0.74	0.013	−1.02	−1.7–−0.3
RSA (x̄) (s)	7.77 (0.14)	7.48 (0.15)	0.29 (0.05)	0.12 to 0.45	<0.001	−1.99	−3.1–−0.9
RSA Best (s)	7.10 (0.14)	7.08 (0.16)	0.02 (0.06)	−0.16 to 0.20	>0.99	0.15	−0.9–0.6
RSA FI (%)	9.4 (1.8)	5.7 (1.1)	3.7 (0.5)	1.92 to 5.48	<0.001	2.47	−3.7–−1.2

SD = Standard deviation; CI = Confidence interval; *d* = Cohen’s d; SE = Standard error; Diff = Difference; RSA = Repeated sprint ability; NMAT = New multi-change of direction agility test; 15 m (ball) = 15 m ball dribbling agility test; FI = Fatigue index.

**Table 4:** Pre vs. Post-test results of the CG (*n* = 10).

	Pre-Test (SD)	Post Test (SD)	Diff (SE)	95% CI of Diff.	*p*-Value	*d*-Value	95% CI (*d*)
5 m (s)	1.11 (0.05)	1.07 (0.06)	0.03 (0.02)	−0.03 to 0.10	0.615	−0.56	−1.4–0.2
10 m (s)	1.89 (0.11)	1.88 (0.10)	0.01 (0.06)	−0.22 to 0.24	>0.99	−0.09	−1.5–1.3
30 m (s)	4.41 (0.29)	4.27 (0.23)	0.12 (0.11)	−0.28 to 0.52	0.970	−0.46	−1.4–0.5
SJ (cm)	27.6 (5.0)	28.7 (3.5)	−1.1 (1.8)	−7.62 to 5.37”	>0.99	0.26	−0.7–1.2
CMJ (cm)	30.4 (4.7)	33.5 (1.8)	−3.1 (1.4)	−8.15 to 2.03	0.429	0.78	−0.1–1.7
15 m (ball) (M/s)	4.83 (0.32)	4.75 (0.30)	0.08 (0.16)	−0.51 to 0.68	>0.99	−0.26	−1.5–0.9
NMAT (s)	9.38 (0.52)	9.61 (0.44)	−0.24 (0.17)	−0.87 to 0.39	0.891	0.49	−0.4–1.3
RSA (x̄) (s)	7.64 (0.16)	7.55 (0.16)	0.09 (0.05)	−0.08 to 0.27	0.539	−0.59	−1.3–0.1
RSA Best (s)	7.02 (0.12)	7.16 (0.13)	0.00 (0.02)	−0.08 to 0.08	>0.99	−0.02	−0.4–0.4
RSA FI (%)	8.9 (1.9)	7.6 (1.2)	1.33 (0.7)	−1.26 to 3.92	0.621	−0.85	−2.0–0.3

SD = Standard deviation; CI = Confidence interval; *d* = Cohen’s d; SE = Standard error; Diff = Difference; RSA = Repeated sprint ability; NMAT = New multi-change of direction agility test; 15 m (ball) = 15 m ball dribbling agility test; FI = Fatigue index.

**Table 5:** Performance Comparison Between EG and CG.

	Change EG (SD)	Change CG (SD)	Diff (SE)	95% CI of Diff.	*p*-Value	*g*-Value	95% CI (*g*)
5 m (s)	0.06 (0.05)	0.03 (0.06)	0.02 (0.02)	−0.02 to 0.07	0.286	−0.43	−1.2–0.4
10 m (s)	0.06 (0.12)	0.01 (0.20)	0.05 (0.06)	−0.08 to 0.18	0.413	−0.33	−1.1–0.5
30 m (s)	0.09 (0.41)	0.12 (0.35)	−0.03 (0.16)	−0.35 to 0.29	0.854	0.07	−0.7–0.8
SJ (cm)	−2.5 (2.9)	−1.1 (5.6)	−1.40 (1.7)	−4.82 to 2.01	0.405	0.33	−0.4–1.1
CMJ (cm)	−3.4 (2.3)	−3.1 (4.4)	−0.38 (1.3)	−3.08 to 2.32	0.776	0.11	−0.7–0.8
15 m (ball) (M/s)	0.15 (0.55)	0.08 (0.51)	0.07 (0.22)	−0.38 to 0.52	0.748	−0.13	−0.9–0.6
NMAT (s)	0.37 (0.41)	−0.24 (0.54)	0.64 (0.19)	0.25 to 1.03	0.002	−1.34	−2.2–−0.5
RSA (x̄) (s)	0.29 (0.20)	0.09 (0.15)	0.19 (0.07)	0.04 to 0.35	0.015	−1.02	−1.8–−0.2
RSA Best (s)	0.02 (0.22)	0.00 (0.07)	0.02 (0.07)	−0.13 to 0.17	0.787	−0.11	−0.9–0.6
RSA FI (%)	3.7 (2.2)	1.33 (2.2)	2.4 (0.9)	0.55 to 4.20	0.013	−1.05	−1.9–−0.2

SD = Standard deviation; CI = Confidence interval; *g* = Cohen’s d; SE = Standard error; Diff = Difference; RSA = Repeated sprint ability; NMAT = New multi-change of direction agility test; 15 m (ball) = 15 m ball dribbling agility test; FI = Fatigue index.

## Data Availability

Data is contained within the article or Supplementary Material.

## Supplementary Materials

The following supporting information can be downloaded at: https://www.mdpi.com/article/10.3390/sports13110394/s1, data; Tests of Homogeneity of Variances.

## Abbreviations

The following abbreviations are used in this manuscript:

U20	Soccer players under the age of 20
RST	Repeated sprint training
LSSG	large-sided soccer games
RCT	Randomised controlled trial
RSA	Repeated sprint ability
NMAT	New Multi-Change of Direction Agility Test
