# Supplementary material for: Effects of Combined Repeated Sprint and Large-Sided Game Training on Physical Performance in Elite U20 Soccer Players: A Randomised Controlled Trial
Source: Sports (Basel). 2025 Nov 5;13(11):394. doi: 10.3390/sports13110394 (PMC12656217; doi:10.3390/sports13110394)
Supplement: Supplementary file 1 [file sports-13-00394-s001.zip › Tests of Homogeneity of Variances.pdf]

Oneway

Tests of Homogeneity of Variances

		Levene Statistic	df1	df2	Sig.
"5M pre"	Based on Mean	1,061	1	24	,313
	Based on Median	,888	1	24	,355
	Based on Median and with adjusted df	,888	1	22,890	,356
	Based on trimmed mean	,990	1	24	,330
"10m pre"	Based on Mean	,246	1	24	,625
	Based on Median	,239	1	24	,630
	Based on Median and with adjusted df	,239	1	23,977	,630
	Based on trimmed mean	,245	1	24	,625
"30M pre"	Based on Mean	,010	1	24	,923
	Based on Median	,005	1	24	,945
	Based on Median and with adjusted df	,005	1	23,827	,945
	Based on trimmed mean	,005	1	24	,942
"SJ pre"	Based on Mean	,143	1	24	,708
	Based on Median	,171	1	24	,683
	Based on Median and with adjusted df	,171	1	23,977	,683
	Based on trimmed mean	,166	1	24	,687
"CMJ pre"	Based on Mean	,149	1	24	,703
	Based on Median	,203	1	24	,656
	Based on Median and with adjusted df	,203	1	23,991	,656
	Based on trimmed mean	,200	1	24	,658

Tests of Homogeneity of Variances

		Levene Statistic	df1	df2	Sig.
"Ag T With B pre"	Based on Mean	,605	1	24	,444
	Based on Median	,661	1	24	,424
	Based on Median and with adjusted df	,661	1	23,787	,424
	Based on trimmed mean	,665	1	24	,423
"NMAT pre"	Based on Mean	,234	1	24	,633
	Based on Median	,151	1	24	,701
	Based on Median and with adjusted df	,151	1	23,246	,701
	Based on trimmed mean	,253	1	24	,619
"Average 6 sprints pre"	Based on Mean	,015	1	24	,902
	Based on Median	,003	1	24	,955
	Based on Median and with adjusted df	,003	1	23,779	,955
	Based on trimmed mean	,009	1	24	,925
Best Time pre	Based on Mean	2,265	1	24	,145
	Based on Median	1,647	1	24	,212
	Based on Median and with adjusted df	1,647	1	23,887	,212
	Based on trimmed mean	2,379	1	24	,136
FI=%speed decrement pre	Based on Mean	,007	1	24	,936
	Based on Median	,007	1	24	,936
	Based on Median and with adjusted df	,007	1	23,992	,936
	Based on trimmed mean	,007	1	24	,936
